# Effects of *Ilex aquifolium* Polyphenols on Cardiovascular, Renal and Liver Structure in a Rat Model of Metabolic Syndrome: A Biochemical and Histological Study

**DOI:** 10.3390/molecules31142487

**Published:** 2026-07-16

**Authors:** Renata Nowaczyk, Piotr Kuropka, Antoni Szumny, Natalia Pachura-Hanusek, Anna Zwyrzykowska-Wodzińska, Krystyna Pogoda-Sewrniak, Robert Kupczyński

**Affiliations:** 1 Department of Animal Physiology and Biostructure, Wroclaw University of Environmental and Life Sciences, Norwida 31, 50-375 Wroclaw, Poland; renata.nowaczyk@upwr.edu.pl (R.N.); piotr.kuropka@upwr.edu.pl (P.K.); 2 Department of Biocatalysis and Food Chemistry, Wroclaw University of Environmental and Life Sciences, Norwida 25, 50-375 Wroclaw, Poland; antoni.szumny@upwr.edu.pl; 3 Department of Environment, Animal Hygiene and Welfare, Wroclaw University of Environmental and Life Sciences, Chełmonskiego 38C, 51-630 Wroclaw, Poland; natalia.pachura@upwr.edu.pl (N.P.-H.); krystyna.pogoda-sewerniak@upwr.edu.pl (K.P.-S.); 4 Division of Pig and Horse Breeding, Wroclaw University of Environmental and Life Sciences, Chełmonskiego 38C, 51-630 Wroclaw, Poland; anna.zwyrzykowska@upwr.edu.pl

**Keywords:** polyphenol fraction, *Ilex aquifolium*, cardiomyopathy, nephropathy, inflammation, oxidative stress, obese Zucker rat

## Abstract

Metabolic syndrome (MetS) is a major driver of cardiovascular disease, renal injury, and hepatic steatosis, largely through chronic low-grade inflammation and oxidative stress. This study evaluated the cardioprotective, nephroprotective, and hepatoprotective potential of a polyphenol-rich fraction (PP) isolated from *Ilex aquifolium* leaves in obese Zucker (*fa*/*fa*) rats—a well-established model of MetS. Male rats (8 weeks old) were randomly assigned to receive either a standard diet (CTRL, n = 8) or the same diet supplemented with 10 mg/kg body weight/day of the polyphenol fraction (PP, n = 8) for 8 weeks. The fraction was dominated by chlorogenic acid (59.33 mg/g), neochlorogenic acid (35.39 mg/g), and rutin (9.23 mg/g). At the end of the experiment, biochemical markers of oxidative stress, inflammation, and metabolic status were assessed, together with detailed histopathological examination of the heart, kidneys, and liver. Supplementation with *Ilex aquifolium* polyphenols significantly increased (*p* < 0.01) total antioxidant status, reduced circulating IL-6 and increased MCP-1 levels. These changes were accompanied by clear cardioprotective effects. However, the intervention also produced organ-specific adverse effects. In the kidneys, polyphenol-treated rats exhibited histopathological features consistent with early-stage nephropathy. In the liver, supplementation exacerbated hepatic steatosis compared to controls. In conclusion, the polyphenol-rich fraction from *Ilex aquifolium* exerts potent antioxidant and anti-inflammatory effects that translate into substantial cardioprotection in obese Zucker rats. Nevertheless, its capacity to worsen renal injury and hepatic steatosis highlights the need for caution and further dose- and duration-dependent studies before considering therapeutic application in metabolic syndrome.

## 1. Introduction

Scientific evidence establishes obesity and metabolic syndrome (MetS) as critical precursors to chronic conditions, particularly in the development of cardiovascular disease, type 2 diabetes, and renal damage [1,2]. Metabolic syndrome (MetS) is a cluster of interconnected abnormalities, specifically abdominal obesity, insulin resistance, hypertension, and dyslipidemia, that significantly elevate cardiovascular risk. While each component independently contributes to pathology, their combined effect exponentially increases the severity and progression of cardiovascular disease [1,3]. Moreover, MetS acts as a primary driver of diabetes and hypertension, markedly increasing the risk of chronic and end-stage renal disease. At the same time, functional alterations in epicardial adipose tissue (EAT) induced by obesity accelerate atherosclerosis through heightened inflammatory signalling [4].

Metabolic syndrome (MetS) primarily causes cardiovascular and renal damage through chronic, low-grade inflammation. This systemic inflammatory state involves the increased production of key pro-inflammatory mediators, such as TNF-α, MCP-1 and IL-6, alongside elevated acute-phase reactants, resulting in profound metabolic dysfunction. In addition to elevated pro-inflammatory adipokines, obesity and metabolic syndrome are driven by chronic systemic oxidative stress. The resulting overproduction of reactive oxygen species (ROS) perpetuates the pro-inflammatory state and directly causes clinical complications such as vascular endothelial dysfunction, atherosclerosis, hepatic steatosis and insulin resistance [5,6]. The overproduction of reactive oxygen species (ROS) exacerbates tissue damage and accelerates the progression of obesity, insulin resistance, atherosclerosis and cardiovascular disease [7,8]. The interplay between oxidative stress and chronic inflammation is a fundamental driver of metabolic syndrome (MetS) [9].

Polyphenols are secondary plant metabolites that are renowned for their wide range of health benefits [10,11]. Though widespread in nature, they are highly concentrated in the *Ilex* genus, particularly in *Ilex paraguariensis*, which is commonly consumed as yerba mate [12]. Phenolic compounds are a broad category of phytochemicals that are classified according to their structural configurations, which dictate their diverse biological activities. While essential for plant defense, polyphenols also offer significant antioxidant, anti-inflammatory, and therapeutic benefits for human health, particularly in the management of metabolic disorders [13,14]. Furthermore, these compounds show immense therapeutic promise in the treatment of cardiovascular disease (CVD) and heart failure, as they act as multi-targeted agents that reduce oxidative stress and inflammation simultaneously [15,16,17]. Consequently, they mitigate cardiac damage by inhibiting fibrosis and hypertrophy, as well as by preventing mitochondrial dysfunction and ROS-induced cardiomyocyte apoptosis [18,19]. In addition, they reduce the formation of atherosclerotic plaques by lowering LDL oxidation and reducing the harmful effects of oxidised LDL (ox-LDL) on endothelial cells [18].

Emerging research suggests that phenolic compounds could be an effective treatment for chronic kidney disease (CKD) [20]. These compounds modulate inflammation and oxidative stress, thereby attenuating disease progression and alleviating CKD-associated complications. This dual approach reduces cardiovascular risk by improving lipid regulation and enhancing vascular health [20]. The efficacy of polyphenols is strictly contingent upon their specific structure and dosage. Consequently, their health-promoting effects are directly related to their unique bioavailability and pharmacokinetic profiles [21]. Although the protective role of *Ilex paraguariensis* against obesity-related diseases is well documented, there is a distinct lack of evidence regarding the impact of *Ilex aquifolium* on obesity-induced inflammation and oxidative stress [22]. Although polyphenols are widely recognized as beneficial bioactive compounds, their biological effects may be organ-specific and influenced by factors such as dose, chemical composition, bioavailability, and metabolic status [21]. Furthermore, the biological activity of polyphenols does not always translate into uniformly beneficial outcomes in vivo, particularly due to their extensive metabolism, variable tissue distribution, and potential interactions with cellular signaling pathways [10,20,21]. Additionally, although *Ilex aquifolium* and *Ilex paraguariensis* belong to the same genus, they differ markedly in phytochemical composition. While *Ilex paraguariensis* is rich in methylxanthines, *Ilex aquifolium* contains higher proportions of phenolic acids, flavonoids, and triterpenoids [23]. These differences may affect their biological activity and tissue-specific effects, justifying independent evaluation of *Ilex aquifolium*.

Based on these findings, we investigated the effect of an intervention involving *Ilex aquifolium*-derived polyphenols on the relationship between risk factors and cardio-renal pathology. Using the obese Zucker (*fa*/*fa*) rat model, which mimics human metabolic syndrome (insulin resistance, hyperlipidaemia and cardiovascular dysfunction), we evaluated the intervention’s impact on oxidative stress, inflammation and structural remodelling of the heart, kidney and liver. Furthermore, we evaluated the efficacy of the treatment in reversing cardiovascular and renal dysfunction, specifically cardiomyopathy and focal segmental glomerulosclerosis, as well as associated haematological alterations [24,25]. Our investigation aimed to evaluate both the potential beneficial and adverse effects of Ilex aquifolium-derived polyphenols on cardiovascular, renal, and hepatic tissues in a rat model of metabolic syndrome.

## 2. Results

### 2.1. Phenolic Enriched Fraction Profile

The phytochemical analysis of the polyphenol-rich fraction obtained from *Ilex aquifolium* leaves revealed the presence of phenolic acids, flavonoids, and organic acids (Table 1). The fraction was dominated by hydroxycinnamic acid derivatives, with chlorogenic acid identified as the major compound (59.33 mg/g d.w.), followed by neochlorogenic acid (35.39 mg/g d.w.) and rutin (9.23 mg/g d.w.). Considerable amounts of cryptochlorogenic acid and dicaffeoylquinic acid isomers were also detected.

Lower concentrations of caffeic acid, ferulic acid, and quercetin were observed. In addition to polyphenolic compounds, the analyzed fraction also contained organic acids, including citric, malic, and quinic acids. The predominance of chlorogenic acid derivatives indicates that the obtained polyphenol-rich fraction may exhibit strong antioxidant activity. However, other phytochemicals naturally present in the fraction may also have contributed to the biological effects observed in the present study.

### 2.2. Animal Model

Table 2 presents the effects of dietary polyphenol fraction supplementation on body weight and feed intake in Zucker rats. Initial body weight was highly comparable between the groups, and the lack of statistical significance confirms proper randomisation and baseline equivalence. A lower final body weight was observed in the polyphenol (PP) group than in the control (CTRL) group (*p* < 0.05). Mean feed intake was slightly reduced in the PP group (119.42 g per week) compared to the CTRL group (124.34 g per week); however, this difference was not statistically significant.

### 2.3. Haematological/Biochemical Analysis

Haematological analysis indicated that dietary polyphenol supplementation had differential effects on various blood parameters in Zucker rats, with findings supporting the overall safety of the intervention (Table 3). Haematological parameters, including white blood cell count (WBC), red blood cell count (RBC) and haemoglobin concentration (HGB), were similar in the CTRL and PP groups (*p* > 0.05). Analysis of the haematological profile showed a significantly higher haematocrit (HCT) value in the PP group than in the CTRL group (*p* < 0.02), with simultaneous stability of red blood cell parameters (MCV, MCH and MCHC). Although the PP group exhibited an upward trend in platelet count (PLT), this increase did not reach statistical significance, largely due to high inter-individual variability.

The PP group exhibited lower glucose concentrations than the CTRL group (Table 4). Conversely, although this variation was not statistically confirmed, insulin concentrations showed a different relationship. Administering polyphenols resulted in a significant reduction in plasma non-esterified fatty acid (NEFA) concentrations (*p* < 0.01), suggesting a potential improvement in lipid metabolism. At the same time, we observed a significant decrease in HDL cholesterol (*p* < 0.01) alongside an increase in total cholesterol concentration. However, the difference in total cholesterol concentration between the two groups failed to reach statistical significance. The PP group exhibited significantly lower albumin levels (*p* < 0.01) and a tendency towards reduced total protein (*p* < 0.01) compared to the CTRL group. Furthermore, polyphenol supplementation significantly reduced inorganic phosphorus levels (*p* < 0.03) and increased creatinine levels (*p* < 0.02) in the PP group, while calcium and magnesium levels remained comparable between groups.

A pronounced effect of the PP fraction was observed in antioxidant status parameters (Figure 1). Supplementation caused a significant increase in total antioxidant status (*p* < 0.01), which confirms the high antioxidant potential of the compounds studied in the applied animal model. Notably, this increase in TAS occurred without any significant changes in glutathione reductase (GR) activity or ROS and NO levels. A modulatory effect was also observed in the immunological profile. Significant decreases in interleukin-6 (*p* < 0.01) and increases in MCP-1 protein levels (*p* < 0.01) were found in the PP group. In contrast, IL-10 levels did not change significantly.

### 2.4. Histopathological Examination

#### 2.4.1. Heart Morphology

Histological evaluation of the CTRL group (Figure 2A,C,E,G) revealed substantial myocardial remodelling. This was evidenced by significant infiltration of mast cells between cardiomyocytes (Figure 2A), as well as accumulation of macrophages and activated fibroblasts around blood vessels and in the interstitium (Figure 2E,G). Furthermore, the CTRL group displayed significant degenerative alterations, including microvascular capillary wall thickening (Figure 2C) and an increased cardiomyocyte cross-sectional area (Figure 2G). This contrasts with the reduced damage observed in polyphenol-treated rats (Figure 2H). The CTRL group exhibited moderate-to-severe, localised degeneration specifically within branched cardiomyocytes, characterised by nuclear pyknosis, vacuolar degeneration and myofibrillar lysis, despite maintaining distinct cross-striations and central nuclei (Figure 2G). Selected cardiomyocytes exhibited basophilic inclusions (Figure 2G, inset).

Histological analysis of the PP group (Figure 2B,D,F,H) revealed that the myofibre architecture was well-preserved (Figure 2B,F). This was characterised by cardiomyocytes with normal branching and distinct central nuclei, as well as minimal interstitial or perivascular fibrosis (Figure 2D,H). The myocardium also exhibited a dense and well-preserved capillary network (Figure 2D,F,H). Compared to control samples, the PP group displayed reduced cardiomyocyte size (Figure 2H) and markedly lower mast cell infiltration (Figure 2B).

#### 2.4.2. Renal Morphology

Examination of the control kidneys revealed mild expansion of the mesangial matrix and mesangial hypercellularity, with no alterations to the podocytes; the glomeruli remained free of sclerosis (Figure 3A). Most glomeruli in the CTRL group exhibited significantly reduced Bowman’s spaces (Figure 3A). Interstitial fibrosis and tubular basement membrane thickening were absent in both the CTRL and PP groups (Figure 3A,C). Stromal alterations were limited to the PP group and featured minimal fibroblast activation and sparse macrophage/lymphocyte infiltration (Figure 3B). Histological analysis revealed glomerular enlargement in the PP group compared to the CTRL group (Figure 3B,D). In the PP group, podocytes surrounding the vascular loops displayed hypertrophy (Figure 3B,D), frequently developing synechiae and associated segmental sclerosis (Figure 3B–D). Most of the glomeruli observed exhibited dilated Bowman’s spaces (Figure 3B,D). Histological evaluation of the PP group revealed features of early-stage nephropathy, with the observed changes being consistent with an initial, pre-nodular stage.

#### 2.4.3. Liver Morphology

Microscopic analysis revealed moderate macrovesicular steatosis in the peripheral parts of the lobules of both the CTRL (Figure 4A) and PP (Figure 4C) groups. This was more pronounced in the PP group, though no severe parenchymal damage was observed. Although portal fibroblast activation occurred in both groups (Figure 4A,D), the CTRL group exhibited more pronounced macrophage activation and scattered perivascular inflammatory foci (Figure 4B) compared to the PP group (Figure 4D).

## 3. Discussion

Emerging research confirms that plant polyphenols are powerful regulators of glycolipid metabolism, with preclinical studies demonstrating their ability to mitigate metabolic dysfunction through antioxidant and anti-inflammatory mechanisms. By modulating critical signalling pathways, including NF-κB, AMPK, and PPARγ, polyphenols effectively optimize metabolic function and combat inflammation-driven metabolic disorders. Furthermore, evidence supports their role in protecting liver health through the regulation of lipid metabolism and oxidative stress [26,27,28]. Additionally, these compounds exhibit dual antioxidant activity by functioning through both direct reactive oxygen species (ROS) scavenging and the upregulation of antioxidant enzymes, particularly superoxide dismutase and catalase [29]. By neutralizing free radicals, these polyphenols protect cellular structures and DNA, mitigating oxidative stress-induced damage and aiding in the prevention of chronic diseases [30]. Nevertheless, these outcomes are not universal and can vary depending on the compound, dosage and experimental model [31,32,33,34]. Our findings confirm that *Ilex aquifolim* polyphenols, due to their high content of chlorogenic acid derivatives and rutin, exhibit strong antioxidant potential. The analyzed polyphenol-rich fraction was dominated by chlorogenic acid, neochlorogenic acid, cryptochlorogenic acid, and dicaffeoylquinic acid isomers, which is consistent with previous reports describing hydroxycinnamic acids as the major phenolic constituents of *Ilex* species, particularly yerba mate [12,35,36]. Similar phytochemical profiles rich in chlorogenic acid derivatives were also reported by Yue et al., who identified these compounds as key contributors to the antioxidant and metabolic regulatory activity of yerba mate extracts [37]. Furthermore, chlorogenic acid-rich extracts have been shown to attenuate oxidative stress, modulate inflammatory pathways, and improve metabolic homeostasis in obesity-related disorders, which corresponds well with the biochemical and histological findings observed in the present study [38,39,40]. It should be emphasized, however, that the tested preparation represented a polyphenol-rich fraction rather than purified individual polyphenols. Therefore, although chlorogenic acid derivatives were the predominant constituents, other naturally occurring phytochemicals present in the fraction, including triterpenoid saponins and other triterpenoids characteristic of *Ilex aquifolium*, may also have contributed to both the beneficial and adverse biological effects observed.

As hallmark models of metabolic syndrome, obese Zucker (*fa*/*fa*) and Zucker Diabetic Fatty (ZDF) rats are widely used to study the progression of obesity, insulin resistance, dyslipidaemia, and adipose tissue inflammation [41,42,43]. Their progression toward a human-like metabolic syndrome makes them an excellent model for studying dietary and pharmacological treatments [44,45,46]. Due to a leptin receptor mutation, these models exhibit hyperphagia and rapid weight gain. Although supplementation with the polyphenol fraction did not significantly affect feed intake, the observed tendency towards lower final body weight suggests that it may modulate metabolic rate or nutrient utilisation efficiency. But supplementation with *Ilex aquifolium* polyphenols did not produce statistically significant changes in glycaemia or insulin levels compared to the control group; it did yield a clinically relevant reduction in free fatty acids (NEFA). The observed reduction in circulating NEFA concentrations may reflect enhanced fatty acid utilization through increased β-oxidation and improved metabolic flexibility, as previously reported for several polyphenol-rich extracts. However, reduced adipose tissue lipolysis cannot be excluded, and the present study does not allow discrimination between these mechanisms. Interestingly, despite lower circulating NEFA concentrations, hepatic steatosis was more pronounced in the PP group, suggesting that plasma NEFA concentrations do not necessarily reflect improved hepatic lipid handling. These findings indicate a complex regulation of lipid metabolism that warrants further investigation. Given the minimal impact on glycaemic and insulinemic parameters, the mechanism of action of the polyphenol fraction may be more closely related to modulation of inflammatory and oxidative pathways than to direct regulation of carbohydrate metabolism [47,48]. Interestingly, despite the reduction in circulating NEFA concentrations, the PP group exhibited more pronounced hepatic steatosis. This observation suggests that reduced plasma NEFA levels do not necessarily reflect improved hepatic lipid metabolism and may indicate altered hepatic lipid handling or accumulation.

Bioactive dietary components, particularly polyphenols, modulate blood parameters, primarily by reducing leukocyte counts and improving inflammatory markers. Our study supports these findings and demonstrates a direct mitigation of the chronic, low-grade inflammation associated with obesity [49,50]. The observed effects are also dependent on the specifics and duration of the intervention, as blood parameters often indicate improved systemic metabolic and oxidative regulation [51]. Concurrent increases in HCT and stable erythrocyte counts point toward subtle modulation of erythropoiesis or minor plasma volume shifts. This aligns with established findings regarding phytobiotic supplementation [52]. Moreover, decreased albumin levels indicate a reduction in hepatic synthesis caused by chronic metabolic stress, while hypophosphataemia suggests disturbances in mineral homeostasis. In rat models, elevated serum creatinine is an indirect indicator of a reduced glomerular filtration rate (GFR), indicating progressive renal dysfunction. This biomarker is particularly important for assessing metabolic and diabetic nephropathy [53]. These relationships were further validated by histological analysis.

Oxidative stress is widely recognised as a critical factor in obesity and insulin resistance, a connection that has been extensively documented in foundational research by Furukawa et al. and Valko et al. [54,55]. Sies indicates that phytochemicals may modulate the antioxidant system by acting as regulators of oxidative stress [56]. Our findings demonstrate a significant increase in total antioxidant status (TAS) following polyphenol fraction supplementation (*p* < 0.01), which validates the strong antioxidant potential of the tested compounds in vivo. Interestingly, this increase occurred without any significant alteration to glutathione reductase (GR) activity, ROS or NO levels. Metabolic dysfunction in Zucker rats is characterised by increased ROS production and decreased NO levels, which are key drivers of the accompanying vascular pathology. Furthermore, the significant increase in MCP-1 protein concentration (1.10 vs. 0.76 ng/mL; *p* < 0.01) suggests the specific activation of monocyte chemotaxis, despite the general anti-inflammatory response evidenced by the decreased IL-6 levels. The reduction in IL-6 levels observed aligns with Hotamisligil’s assertion that this cytokine plays a pivotal role in the low-grade inflammation that drives obesity and insulin resistance [57]. Concurrently, the upregulation of MCP-1 indicates a noteworthy immunomodulatory effect. MCP-1 plays an essential dual role in mediating inflammation and tissue repair by recruiting monocytes [58]. The absence of significant changes in IL-10 suggests that the beneficial effects observed in the polyphenol fraction are mediated by pro-inflammatory pathways rather than anti-inflammatory regulation. This finding in the Zucker model may represent an adaptive tissue mechanism in response to chronic metabolic stress. In line with the existing literature, these results prove that the biological effects of polyphenols are largely due to the modulation of specific signalling cascades, including those involved in responses to oxidative stress, anti-inflammatory processes and apoptosis [59].

Chronic low-grade inflammation and elevated oxidative stress are major drivers of the development of diabetic cardiomyopathy (DCM) [60]. Histologically, animals in the CTRL group exhibited the following characteristic features of DCM: structural remodeling; minor and moderate interstitial and perivascular fibrosis; cardiomyocyte hypertrophy; and increased myocardial cell death [61]. Basophilic degeneration, characterised by the accumulation of glycosylated amyloid precursor protein fragments in the myocardium, was present in the CTRL group. While these inclusions are a common part of the aging process, their severity is strongly linked to metabolic syndrome and associated myocardial fibrosis [62]. Contrary to the expected structural changes associated with diabetic cardiomyopathy, such as fibrosis and triglyceride accumulation, the PP group showed no such alterations, even in the context of insulin-resistant hyperinsulinaemia [63,64,65,66]. A novel finding of the present study is that the cardioprotective effects of the tested polyphenol fraction occurred simultaneously with adverse histopathological changes in the kidneys and liver. Our findings suggest that the cardiomyocytes in the PP group were protected from the structural degradation of contractile proteins typically observed in cases of hypertrophy or chronic heart failure. Histopathological evaluation demonstrated that heart tissue from the PP group showed improved structural changes, specifically reduced nuclear/cytoplasmic alterations, diminished inflammatory infiltration and a transition to inactive, flattened fibroblasts, relative to control samples. In line with studies on the pathophysiology of diabetic cardiomyopathy, our research confirms a strong correlation between imaging-detected cardiomyocyte remodelling and elevated mast cell activation [67,68,69].

Consistent with previous studies, our research confirms that polyphenols provide strong cardioprotective effects by inhibiting structural remodelling [16,70]. They suppress myocardial cell hypertrophy, thereby limiting the pathological compensatory mechanisms driven by diabetes or oxidative stress. Our findings suggest that polyphenols prevent oxidative injury, which signals heart cells to grow, by reducing the production of reactive oxygen species (ROS) and enhancing antioxidant defences [71]. Furthermore, our research provides robust evidence that these compounds act as potent modulators of inflammatory pathways, successfully mitigating the chronic inflammation that accelerates cardiac hypertrophy and fibrosis [16,72].

Chronic kidney disease (CKD) is closely linked to metabolic syndrome, diabetes, and cardiovascular disease, with oxidative stress acting as a central mechanism driving disease progression [73]. Pathologically, CKD is characterised by glomerulosclerosis, tubular atrophy, interstitial fibrosis, vascular remodelling, and diminished renal regenerative capacity. Furthermore, inflammation driven by oxidative stress accelerates atherosclerosis, serving as a critical driver for both CKD and the cardiorenal syndrome observed in this study [20]. The obese Zucker rats (*fa*/*fa*) are a widely utilised model for metabolic syndrome and type 2 diabetes, exhibiting profound insulin resistance and hyperlipidaemia. These metabolic perturbations, which correlate with liver histology and blood biomarkers—especially within the PP group—drive progressive renal damage, characterised by mesangial expansion, macrophage infiltration and fibrosis, leading to focal segmental glomerulosclerosis (FSGS) [74,75]. Despite the absence of prominent tubular, interstitial, or arteriolar lesions, the PP group exhibited clear signs of developing early-stages nephropathy, including proteinuria and impaired glomerular filtration rates. Furthermore, this group exhibited significant, characteristic structural alterations, primarily localized within the glomerulus [76]. The clinical manifestations of early-stage nephropathy are strongly correlated with these underlying structural changes, particularly the extent of mesangial expansion observed in the CTRL and PP groups [77]. Chronic glomerular hyperfiltration drives compensatory hypertrophy and subsequent glomerulosclerosis, leading to progressive and irreversible renal dysfunction, as well as elevated serum creatinine levels. In line with previous studies, our results confirm that podocyte hypertrophy occurs as a compensatory mechanism in high-volume states such as obesity and diabetes. This is evidenced by simultaneous glomerular basement membrane thickening and increased mesangial matrix expansion [78,79]. This response is typical of the pathological progression observed in obesity-related glomerulopathy, diabetic nephropathy and focal segmental glomerulosclerosis (FSGS) [80,81].

Growing evidence points to the renal-protective potential of polyphenols, yet translating this into clinical practice requires careful consideration [82]. While preliminary findings suggest that certain polyphenols could mitigate nephrotoxic damage, further research is needed to confirm their efficacy and safety in humans. Due to extensive metabolism, in vitro success rarely reflects the pharmacokinetic realities of the human body [82,83]. Unlike most studies, which have a purely positive focus, our research aligns with findings that highlight the potential adverse effects of polyphenols on renal and hepatic health, as evidenced by histological images and blood test results [83]. Whereas most research emphasises the beneficial impact of polyphenols, our study demonstrates the opposite, showing that polyphenols can impair renal function and structure [82]. Chronic hyperglycaemia damages the renal glomeruli, initially causing hyperfiltration (an increased glomerular filtration rate, or GFR). Specifically, elevated creatinine levels combined with hypoalbuminaemia and reduced total protein indicate disruption to the glomerular filtration barrier. Although nephrotoxicity is typically characterised by reduced GFR and oliguria, our studies revealed elevated creatinine levels alongside significant polyuria. Furthermore, elevated MCP-1 drives renal tissue remodelling and fibrosis through macrophage recruitment, with histological changes aligning with the altered biochemical profile of the experimental group (PP) [84]. Because only a single dose of the polyphenol-rich fraction was evaluated, we cannot exclude that the observed renal and hepatic alterations were dose-dependent. Moreover, gut microbiota-mediated biotransformation of polyphenols and their metabolites, as well as potential alterations in bile acid metabolism and hepatic lipid handling, may have contributed to the organ-specific effects observed [21,85,86]. Since neither gut microbiota composition nor bile acid metabolism was investigated in the present study, these mechanisms remain speculative and should be addressed in future dose–response and mechanistic studies.

Importantly, the present study demonstrates that the biological effects of the Ilex aquifolium polyphenol-rich fraction are organ-specific. While supplementation improved antioxidant status, reduced IL-6 concentrations, and exerted marked cardioprotective effects, it was also associated with histopathological alterations in the kidneys and increased hepatic steatosis. These findings provide novel evidence that the health effects of polyphenols in metabolic syndrome may not be uniformly beneficial across organs and emphasize the importance of comprehensive multi-organ evaluation. A limitation of this study is the use of a single dose level, which precludes a detailed assessment of dose–response relationships and the determination of potential therapeutic and adverse-effect thresholds. Therefore, the observed effects should be interpreted with caution. In addition, the absence of urinary biomarkers limits the functional assessment of renal status. Another limitation of this study is that the tested preparation represented a polyphenol-rich fraction rather than purified polyphenols. Therefore, the contribution of other bioactive constituents naturally present in *Ilex aquifolium,* including triterpenoids and other phytochemicals, cannot be excluded. Furthermore, residual extraction solvent was not analytically quantified, although methanol was removed under reduced pressure prior to biological testing. Future studies should compare purified compounds with aqueous and hydroalcoholic extracts and evaluate the contribution of individual phytochemical classes. Future studies incorporating multiple dose levels and additional renal function markers would help to further clarify the biological effects and safety profile of the tested polyphenol fraction. Furthermore, neither gut microbiota composition nor bile acid metabolism was evaluated, preventing mechanistic interpretation of the observed organ-specific effects.

## 4. Materials and Methods

### 4.1. Plant Material and Isolation of the Ilex aquifolium Polyphenols

Fresh plant material in the form of *I. aquifolium* leaves was obtained from the Vegetable and Ornamental Plant Research and Education Station at the University of Life Sciences’ own cultivation (Psary, Poland). The holly shrubs were cultivated in the ornamental plant collection at the Department of Horticulture. After harvesting, the leaves were cleaned and freeze-dried for 24 h using a Lyovac GT 2 apparatus. The lyophilised leaves were then crushed to obtain a homogeneous material.

### 4.2. Analysis of the Polyphenol-Rich Fraction (PP)

Powdered, dried *Ilex aquifsolium* leaves (20 g) were subjected to two successive extractions with methanol. In each extraction cycle, the plant material was combined with 100 mL of methanol (Sigma–Aldrich, Darmstadt, Germany) in a glass vessel and agitated for 24 h at room temperature. After the first supernatant had been collected, the remaining plant material was extracted again under the same conditions using a fresh 100 mL portion of solvent. The two supernatants were centrifuged at 13,000 rpm for 10 min, filtered, pooled, and concentrated under reduced pressure in a rotary evaporator to remove methanol before biological testing. Methanol was used because the purpose of the procedure was to obtain an analytically characterised phenolic fraction for experimental studies rather than food-grade preparation. For LC–MS/MS analysis, the resulting concentrate was diluted 200-fold with the appropriate solvent system.

The phenolic constituents of the PP fraction were identified and quantified by LC–MS/MS. The analytical platform comprised a Shimadzu Prominence-i LC-2030C high-performance liquid chromatograph connected to a Shimadzu LCMS-8045 triple-quadrupole mass spectrometer (Shimadzu, Kyoto, Japan). Chromatographic separation was performed at 40 °C on a Luna C18 column (100 Å, 150 mm × 2.1 mm × 3 μm; Phenomenex, Torrance, CA, USA). The eluent system consisted of 0.1% formic acid in water (A) and acetonitrile (B), with both solvents being of LC–MS grade (>99.95% purity). The flow rate was set at 0.35 mL min^−1^. The gradient started at 10% B, reached 20% B after 5 min and 60% B after 10 min, and then returned to 10% B at 13 min; this composition was maintained until 17 min. A 10 μL aliquot was injected for each run, and every sample was analysed in triplicate.

Analytes were detected in negative electrospray ionisation mode (ESI−) using multiple reaction monitoring (MRM). The capillary voltage was 4.0 kV. Compound-specific MRM transitions and acquisition conditions are summarised in Table 5. The remaining source parameters were as follows: nebulising gas, 3 L min^−1^; heating gas, 10 L min^−1^; drying gas, 10 L min^−1^; interface temperature, 300 °C; desolvation-line temperature, 274 °C; and heat-block temperature, 400 °C.

Analyte concentrations were calculated from external calibration curves generated with authentic reference standards. Data were acquired and processed in LabSolutions Postrun Analysis software ver. 5.120 (Shimadzu, Kyoto, Japan). Supplementary Table S1 contains the full analytical dataset, including compound assignments, retention times, MRM transitions, and quantitative results.

### 4.3. Animal and Experimental Design

All procedures and manipulations performed on living animals were in accordance with the decision of the Local Ethics Committee (no. 09/2019/P1, Wroclaw, Poland) and the associated guidelines of the European Communities Council (no. 86/609/EEC), which governs animal protection. The experimental design was based on the limitations imposed by the 3R rule.

This study used obese Zucker (*fa*/*fa*) rats (OZRs) as a model of metabolic syndrome (MetS), due to their well-established use in nutritional and biochemical studies involving dietary supplementation with bioactive plant components. Six-week-old obese Zucker male rats (n = 16) were purchased from Charles River Laboratories International, Inc., Wilmington, MA, USA, and fed a standard diet for two weeks to acclimatise them to their new environment. The animals were housed in pairs under controlled conditions, with a constant indoor temperature of 22 ± 1 °C, relative air humidity of 50–70%, a 12 h light/dark cycle, and air changes in the room. The rats were fed ad libitum with free access to tap water. The animals were fed commercial pellets containing 16% protein, 2.5% fat, 7.5% fibre, and supplemented vitamins and minerals, according to AOAC methods [87].

After acclimatisation, eight-week-old rats were subjected to the following dietary treatments: a standard diet (CTRL group, n = 8) or a standard diet to which 10 mg/kg body weight of the isolated polyphenol fraction acquired from *Ilex aquifolium* leaves was added (PP group, n = 8). The polyphenol extracts were administered to the animals individually per os. The selected dose (10 mg/kg BW/day) was based on our previous in vitro studies, including cytotoxicity assessments performed on mammalian cell lines (including Caco-2 cells), which demonstrated good safety and biological activity of the polyphenol fraction. The dose selection was further supported by previously published experimental studies evaluating the biological effects of polyphenols in animal models [88,89,90].

The body weight of the rats was monitored twice weekly, while their diet was checked daily. Highly trained staff and a veterinarian properly monitored and observed the animals, taking their well-being and health parameters into account. The experiment lasted eight weeks. After blood was collected at the end of the experiment, the rats were euthanised by cervical dislocation. Selected internal organs and tissue samples were then fixed for histological analysis.

### 4.4. Haemathological and Biochemical Analysis

Blood samples were collected at 10:00 a.m., approximately 4–5 h after feeding and immediately before euthanasia. Whole blood for haematological analysis was collected into sterile EDTA-K3 tubes (Sarstedt, Nümbrecht, Germany) and analysed immediately using an ABC Vet analyser (Horiba ABX Diagnostics, Montpellier, France). The evaluated haematological parameters included red blood cells (RBC), white blood cells (WBC), platelets (PLT), haemoglobin (HGB), haematocrit (HCT), mean corpuscular volume (MCV), mean corpuscular haemoglobin (MCH), mean corpuscular haemoglobin concentration (MCHC), lymphocytes (LYM), monocytes (MON), and granulocytes (GRA). Whole blood for glutathione reductase (GR) determination was collected into dedicated EDTA microtubes (VT-100 STK, CML, Nemours, France).

Blood intended for biochemical and serum-based analyses was collected into serum tubes containing a clot activator (Sarstedt, Nümbrecht, Germany). After allowing the blood to clot, samples were centrifuged within 2 h of collection at 3000× *g* for 10 min at room temperature, and the separated serum was stored at −20 °C until analysis. Biochemical parameters were determined using a Pentra 400 analyser (Horiba ABX Diagnostics, Montpellier, France). Non-esterified fatty acids (NEFAs) were measured enzymatically using Randox reagents (Crumlin, Dublin, Ireland). Triglycerides (TG), total cholesterol, high-density lipoprotein cholesterol (HDL), low-density lipoprotein cholesterol (LDL), glucose, aspartate aminotransferase (AST), total protein (TP), and albumin (Alb) were analysed using HORIBA ABX reagents (Montpellier, France) according to the manufacturer’s instructions.

The serum total antioxidant capacity (TAC) was determined using a colorimetric ABTS [2,2′-azino-bis(3-ethylbenzothiazoline-6-sulfonic acid)] assay based on a peroxidase reaction. Glutathione reductase (GR) activity was measured spectrophotometrically in whole blood using an NADPH-dependent assay by monitoring the decrease in absorbance at 340 nm during the reduction of oxidized glutathione (GSSG). Serum concentrations of monocyte chemoattractant protein-1 (MCP-1), reactive oxygen species (ROS), and nitric oxide (NO) were determined using commercially available assay kits (MyBioSource, San Diego, CA, USA). MCP-1 and ROS were quantified using sandwich enzyme-linked immunosorbent assays (ELISA) with horseradish peroxidase (HRP)-based colorimetric detection, whereas NO concentration was measured colorimetrically by quantifying total nitrite/nitrate (NO_2_^−^/NO_3_^−^) at 550 nm. All measurements were performed using a Synergy multi-mode microplate reader (BioTek Instruments, Winooski, VT, USA).

### 4.5. Histopathological Tissue Examination

The tissue samples were fixed in a 4% buffered formaldehyde solution with a pH of 7.2–7.4 for 48 h, after which they were rinsed in running water for a further 24 h. The material was then dehydrated in a series of alcohols, embedded in paraffin, and processed for histology. The tissue samples were cut into 7 µm thick slides and stained with haematoxylin and eosin (H&E) as standard. Material analysis was performed using a Nikon Eclipse 80i microscope equipped with a Jenoptik Gryphax^®^ Kapella camera and the corresponding Gryphax^®^ software ver. 2.1.0 (JENOPTIK Optical Systems GmbH, Jena, Germany). The histopathological evaluation was conducted by one experienced veterinary pathologist who was unaware of the animals’ group allocation (double-blind study). The assessment included an evaluation of both the incidence and severity of morphological alterations in the heart, kidneys, and liver. The histopathological analysis encompassed the following parameters:

- Cardiovascular system: evaluation of inflammatory infiltrates, fibrosis, and cardiomyocyte structure including hypertrophy.
- Kidneys: assessment of glomerular structural alterations (e.g., hypertrophy, mesangial expansion, and podocyte hypertrophy), glomerulosclerosis, and stromal changes.
- Liver: evaluation of inflammatory infiltrates, fibrosis, and hepatic steatosis.

Multiple representative fields of view were examined for each sample. The morphological alterations were graded and interpreted in accordance with standard histopathological criteria for tissue damage associated with metabolic syndrome.

### 4.6. Statistical Analysis

Statistical analyses were performed using Statistica version 13.3 (StatSoft, Kraków, Poland). Prior to data analysis, the assumptions underlying the applied statistical models were verified. The normality of variable distributions as well as model residuals was evaluated with the Shapiro–Wilk test. All in vivo analyses were performed in duplicate, with group data representing the mean of eight animals (n = 8). The normality of data distribution and model residuals was assessed using the Shapiro–Wilk test. Data are presented as mean ± SEM. Differences between the CTRL and PP groups were analyzed using Student’s *t*-test for independent samples. Data are presented as means and SEM; *p* < 0.05 was considered statistically significant.

## 5. Conclusions

Studies indicate that the polyphenol fraction of *Ilex aquifolium* has comprehensive protective effects on obese Zucker rats, specifically enhancing key biochemical parameters and maintaining cardiac structural integrity. Findings include increased antioxidant capacity, decreased IL-6 concentrations and reduced myocardial mast cells, demonstrating that polyphenol administration effectively mitigates oxidative stress and inflammation. Simultaneously reducing NEFA and glucose levels while increasing insulin sensitivity indicates a reduction in lipotoxicity, corresponding to improved cardiomyocyte morphology in the PP group. The observed increase in MCP-1 indicates the initiation of chemotactic signalling, suggesting active tissue remodelling or early repair processes. Biochemical findings, including hypoalbuminaemia, hypoproteinaemia (decreased total protein) and increased creatinine paired with decreased phosphorus, suggest the coexistence of early glomerular injury and tubular dysfunction in the PP group. This profile is consistent with the studied model and demonstrates that polyphenol supplementation is ineffective at mitigating structural alterations induced by the underlying genetic defect.

In summary, polyphenols have significant cardioprotective effects, mitigating oxidative stress and inflammation. However, their efficacy in halting the progression of genetically determined nephropathy in the Zucker rat model is limited. Simultaneously, supplementation exacerbated fatty liver disease, suggesting that its impact on liver structure is complex rather than uniformly beneficial. To gain a comprehensive understanding of the impact of *Ilex aquifolium* polyphenols, future studies must prioritise long-term dose–response investigations alongside a detailed analysis of liver lipid metabolic pathways and renal safety.

## Figures and Tables

**Figure 1:**
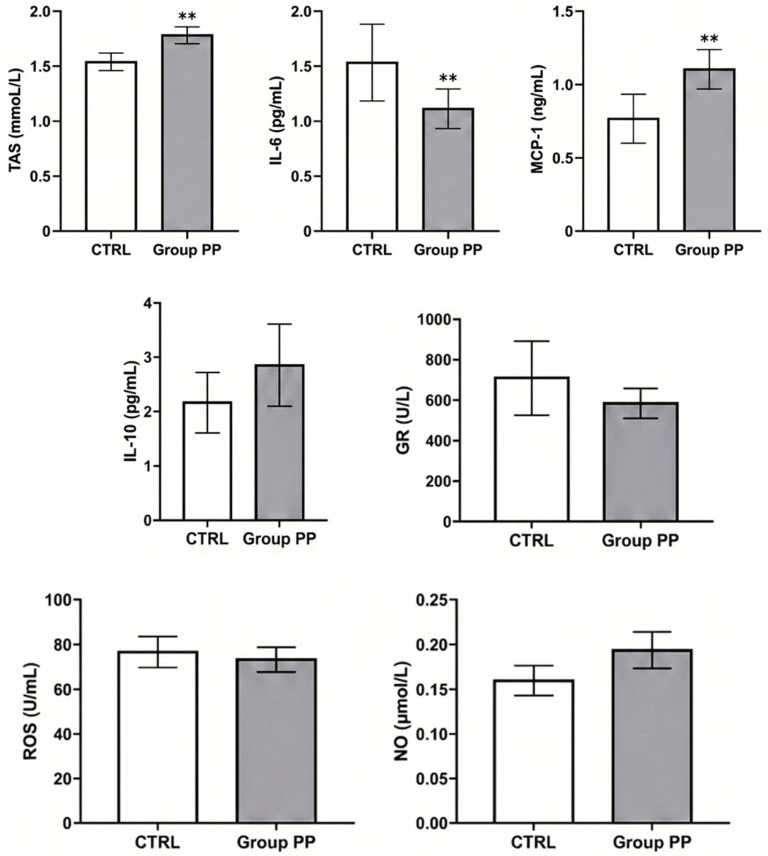
Comparative analysis of oxidative stress markers and inflammatory cytokine levels between CTRL and PP groups in obese Zucker Rats. Statistical evaluation using ANOVA, followed by a post hoc test, revealed statistically significant differences (**; *p* < 0.01) in selected parameters.

**Figure 2:**
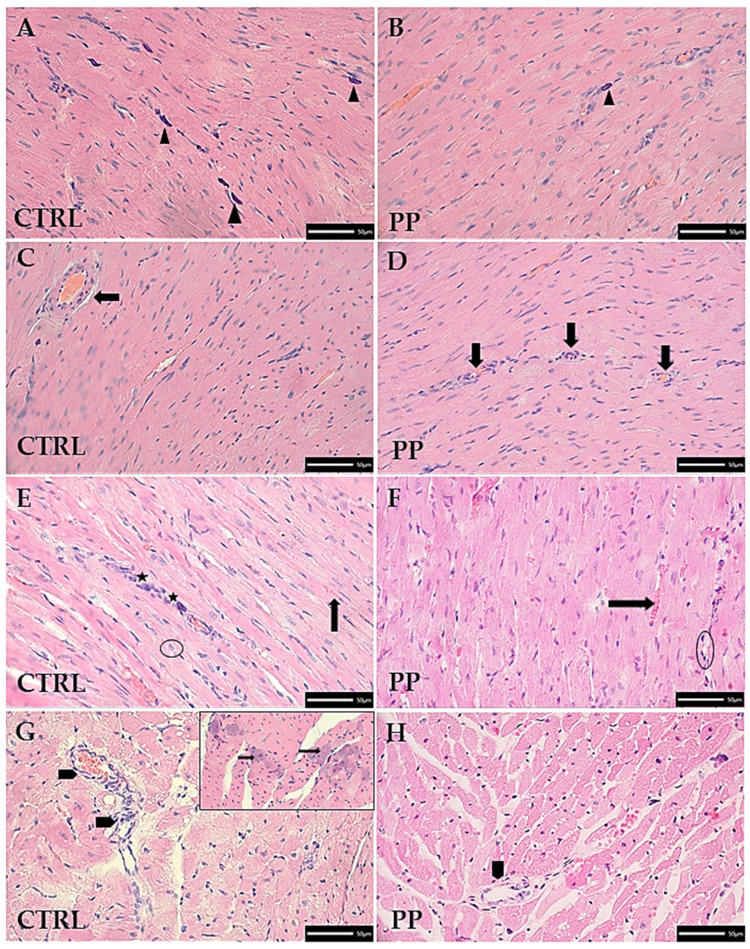
H&E staining of rat heart tissue. Photomicrographs comparing the control (CTRL) group (**A**,**C**,**E**,**G**) with the polyphenol-treated (PP) group (**B**,**D**,**F**,**H**). (**A**,**B**) Longitudinal sections, revealing the structure of the cardiomyocytes and the infiltration of mast cells (arrowhead) in the connective tissue stroma. (**C**,**D**) Representative blood vessel (arrow). (**E**,**F**) Fibroblast morphology highlighting activated cells ((**E**), circle) and flat, inactive cells (**F**), circle), as well as inflammatory cell infiltration ((**E**), asterisk). (**F**,**H**) Normal cardiomyocytes with intact central nuclei and unaffected blood vessels (arrows). (**G**,**H**) Perivascular fibrosis (arrowhead). (**G**) Cardiomyocyte hypertrophy and basophilic inclusion (inset). Mag. 400×. Scale bar: 50 µm (**A**–**H**).

**Figure 3:**
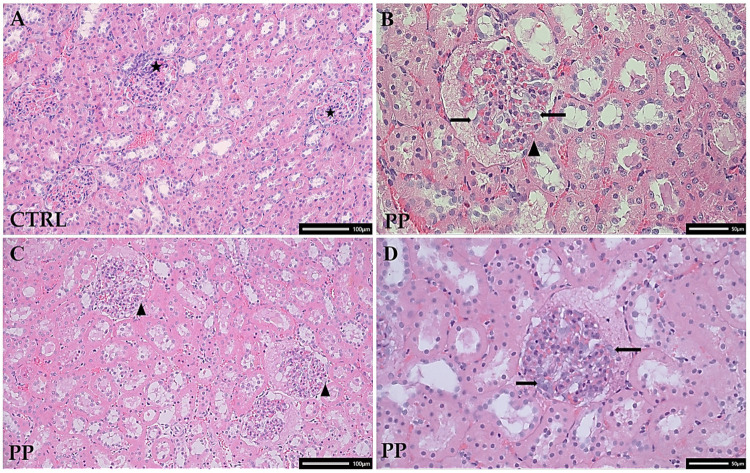
Representative haematoxylin and eosin (H&E)-stained renal tissue showing glomerular lesions in the control (CTRL) group (**A**) and the PP group (**B**–**D**). (**A**) The control sample exhibited segmental increases in glomerular matrix and moderate-to-marked diffuse mesangial cell hypercellularity (asterisk). The PP group (**B**,**D**) demonstrated glomerular structural alterations, including podocyte hypertrophy (arrow) and parietal epithelial cell hyperplasia. (**C**,**D**) The PP group exhibited segmental sclerosis of the capillary tuft with synechia formation (arrowhead) characterised by adhesion to Bowman’s capsule, thickening, wrinkling and localised cellular proliferation. Magnification: 200× ((**A**,**C**); scale bar: 100 µm); 400× ((**B**,**D**); scale bar: 50 µm).

**Figure 4:**
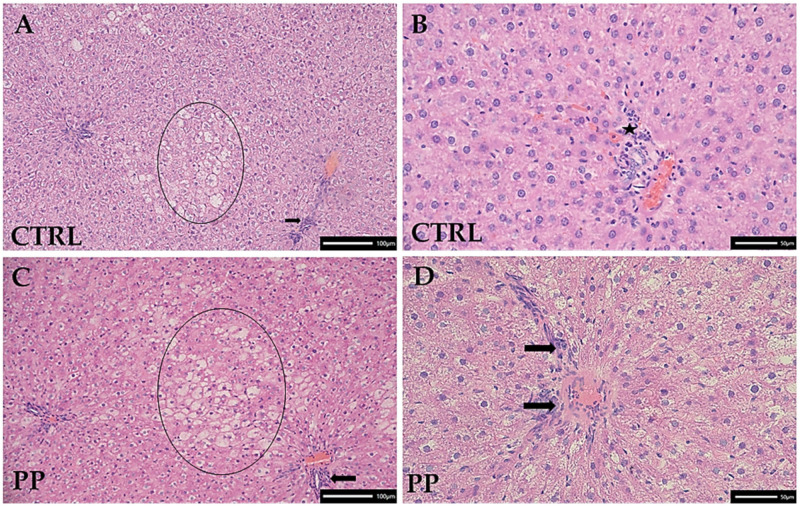
Histopathological analysis of rat livers using H&E staining. (**A**,**B**) Control group: The liver tissue exhibited normal architecture, with moderate steatosis indicated (circle) and mild periportal lymphatic infiltration (asterisk). (**C**) PP group: Hepatocytes displayed increased fat droplets and intracytoplasmic vacuoles (circle). (**A**) Control group and (**D**) experimental group: Portal fibroblast activation (arrow). Magnification: 200× ((**A**,**C**); scale bar: 100 µm); 400× ((**B**,**D**); scale bar: 50 µm).

**Table 1:** Composition of the polyphenol-rich fraction obtained from *Ilex aquifolium* leaves.

Compound	*Ilex aquifolium*
	Concentration (mg/g) d.w.
Citric acid	1.58
Malic acid	3.13
Quinic acid	2.71
Caffeic acid	1.19
Ferulic acid	0.62
Chlorogenic acid	59.33
Cryptochlorogenic acid	5.89
Neochlorogenic acid	35.39
3,5-Dicaffeoylquinic acid	3.87
4,5-Dicaffeoylquinic acid	2.09
Rutin	9.23
Quercetin	1.77

**Table 2:** Body weight and feed intake.

	Group		SEM	*p* < Value
	CTRL ^1^	PP ^2^		
Initial body weight [g]	295.86	296.29	23.87	0.21
Final body weight [g]	524.07	503.45	18.34	0.05
Feed intake [g/week]	124.34	119.42	14.56	0.17

^1^ Control (CTRL) group; ^2^ PP—rats fed a standard diet with the addition of polyphenol fraction.

**Table 3:** Haematological parameters in obese Zucker rats following supplementation with dietary polyphenols.

	Group ^1^		SEM	*p* < Value ^4^
	CTRL ^2^	PP ^3^		
WBC ^5^ G/L	11.23	11.84	0.48	0.55
RBC ^6^ T/L	7.28	7.80	0.15	0.12
HGB ^7^ mmol/L	8.89	9.43	0.16	0.15
HCT ^8^ L/L	0.35	0.38	0.02	0.02
MCV ^9^ fL	48.75	48.71	0.22	0.94
MCH ^10^ fmoL	1.23	1.21	0.01	0.44
MCHC ^11^ mmol/L	25.11	24.83	0.18	0.46
PLT ^12^ G/L	589.63	701.86	61.24	0.39

^1^ Values are mean; ^2^ Control (CTRL) group; ^3^ PP—rats fed a standard diet with the addition of polyphenol fraction; ^4^ Significant differences between groups (ANOVA followed by the post hoc test); ^5^ WBC, white blood cell; ^6^ RBC, red blood cell; ^7^ HGB, haemoglobin; ^8^ HCT, haematocrit; ^9^ MCV, mean corpuscular volume; ^10^ MCH, mean corpuscular haemoglobin; ^11^ MCHC, mean corpuscular haemoglobin concentration; ^12^ PLT, platelets.

**Table 4:** The modulatory effects of dietary polyphenols on biochemical parameters in the blood serum of obese Zucker rats.

	Group ^1^		SEM	*p* < Value ^4^
	CTRL ^2^	PP ^3^		
Biochemical Parameters				
Glucose mmol/L	4.71	3.88	0.25	0.11
Insulin pg/mL	885.86	1054.25	97.51	0.51
Chol. ^5^ mmol/L	4.63	5.13	0.13	0.089
HDL ^6^ mmol/L	2.53	2.09	0.03	0.01
LDL ^7^ mmol/L	2.11	3.04	0.09	0.06
TG ^8^ mmol/L	3.29	4.58	0.33	0.081
NEFA ^9^ mmol/L	2.81	2.04	0.10	0.01
TP ^10^ g/L	86.62	81.17	1.53	0.01
Alb. ^11^ g/L	38.67	36.57	0.39	0.01
Creatinine µmol/L	24.85	26.08	1.12	0.02
Ca mmol/L	2.95	2.99	0.02	0.463
Mg mmol/L	1.07	1.03	0.02	0.320
P mmol/L	4.30	3.65	0.13	0.034

^1^ Values are mean; ^2^ Control (CTRL) group; ^3^ PP—rats fed a standard diet with the addition of polyphenol fraction; ^4^ Significant differences between groups (ANOVA followed by the post hoc test);^5^ Chol., cholesterol; ^6^ HDL, high-density lipoprotein; ^7^ LDL, low-density lipoprotein; ^8^ TG, triglycerides; ^9^ NEFA, non-esterified fatty acids; ^10^ TP. total protein; ^11^ Alb., albumin.

**Table 5:** Multiple reaction monitoring (MRM) transitions and conditions of mass spectrometers.

Compound	Precursor *m*/*z* [M − H]^−^	MRM Product *m*/*z*	Q_1_ Pre Bias (V)	Collision Energy	Q_3_ Pre Bias (V)
Citric acid	191.40	110.95	21.0	12.0	20.0
		86.95	13.0	17.0	17.0
		85.00	13.0	17.0	16.0
Malic acid	133.40	114.90	14.0	16.0	23.0
		71.00	14.0	15.0	12.0
Quinic acid	191.20	85.25	14.0	21.0	30.0
		147.15	14.0	11.0	24.0
		93.10	15.0	23.0	29.0
Caffeic acid	301.20	151.00	22.0	21.0	15.0
		179.00	21.0	18.0	11.0
		121.00	22.0	27.0	24.0
Ferulic acid	193.40	134.00	12.0	14.0	26.0
		177.95	12.0	15.0	30.0
		149.05	12.0	13.0	14.0
Cryptochlorogenic acid	353.00	191.30	17.0	15.0	19.0
		85.00	16.0	43.0	15.0
		93.05	17.0	46.0	17.0
Neochlorogenic acid	353.00	191.30	17.0	15.0	19.0
		85.00	16.0	43.0	15.0
		93.05	17.0	46.0	17.0
Chlorogenic acid	353.00	191.30	17.0	15.0	19.0
		85.00	16.0	43.0	15.0
		93.05	17.0	46.0	17.0
3,5-Dicaffeoylquinic acid	515.00	353.25	24.0	16.0	24.0
		191.30	24.0	31.0	18.0
		179.30	24.0	30.0	17.0
4,5-Dicaffeoylquinic acid	515.30	353.30	24.0	18.0	12.0
		179.10	24.0	30.0	17.0
		191.35	40.0	34.0	11.0
Rutin	609.30	300.15	30.0	39.0	19.0
		301.10	22.0	30.0	19.0
		271.25	22.0	55.0	17.0

## Data Availability

The raw data supporting the conclusions of this article will be made available by the authors on request.

## Supplementary Materials

The following supporting information can be downloaded at: https://www.mdpi.com/article/10.3390/molecules31142487/s1. Table S1: Calibration equations and coefficients of determination (R^2^) of standards used for LC-MS quantification. Figure S1. LC-MS chromatogram of chlorogenic acid detected in Ilex aquifolium leaf extract; Figure S2. LC-MS chromatograms of 3,5-dicaffeoylquinic acid and 4,5-dicaffeoylquinic acid detected in Ilex aquifolium leaf extract; Figure S3. LC-MS chromatogram of rutin detected in Ilex aquifolium leaf extract; Figure S4. LC-MS chromatograms of ferulic acid, quinic acid, citric acid, malic acid, and quercetin detected in Ilex aquifolium leaf extract.
