# Supplementary material for: Effects of Ilex aquifolium Polyphenols on Cardiovascular, Renal and Liver Structure in a Rat Model of Metabolic Syndrome: A Biochemical and Histological Study
Source: Molecules. 2026 Jul 16;31(14):2487. doi: 10.3390/molecules31142487 (PMC13415443; doi:10.3390/molecules31142487)
Supplement: Supplementary file 1 [file molecules-31-02487-s001.zip › molecules-4367117-supplementary.pdf]

Table S1. Calibration equations and coefficients of determination (R^2) of standards used for LC-MS quantification.

Compound	Calibration equation	R^2
Citric acid	$y = 1.672 \times 10^6x - 1.875 \times 10^6$	0.9944
Malic acid	$y = 5.182 \times 10^5x - 3.416 \times 10^5$	0.9992
Quinic acid	$y = 7.996 \times 10^5x + 5.966 \times 10^4$	0.9947
Caffeic acid	$y = 2.034 \times 10^7x + 5.637 \times 10^5$	0.9957
Ferulic acid	$y = 1.587 \times 10^6x + 3.580 \times 10^5$	0.9859
Chlorogenic acid<br>Cryptochlorogenic acid<br>Neochlorogenic acid	$y = 4.006 \times 10^5x + 8.600 \times 10^6$	0.9959
3,5-Dicaffeoylquinic acid	$y = 1.837 \times 10^6x + 2.800 \times 10^4$	0.9993
4,5-Dicaffeoylquinic acid	$y = 8.235 \times 10^5x + 8.003 \times 10^5$	0.9982
Rutin	$y = 8.532 \times 10^5x + 2.316 \times 10^5$	0.9976
Quercetin	$y = 9.248 \times 10^6x + 4.552 \times 10^3$	0.9997

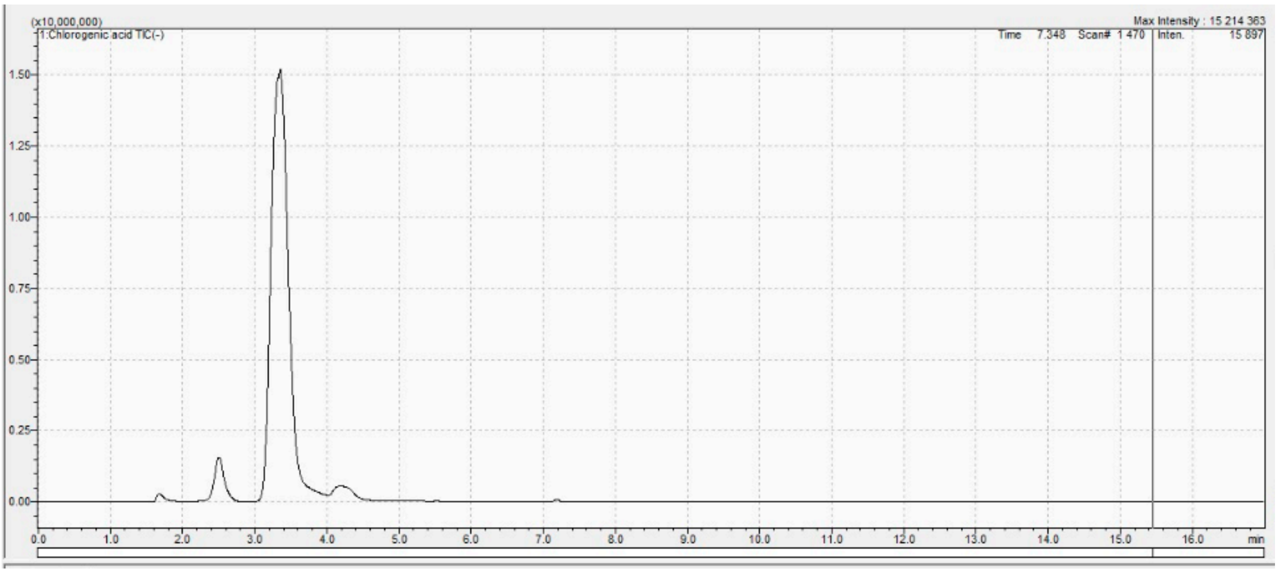

Figure S1. LC-MS chromatogram of chlorogenic acid detected in *Ilex aquifolium* leaf extract.

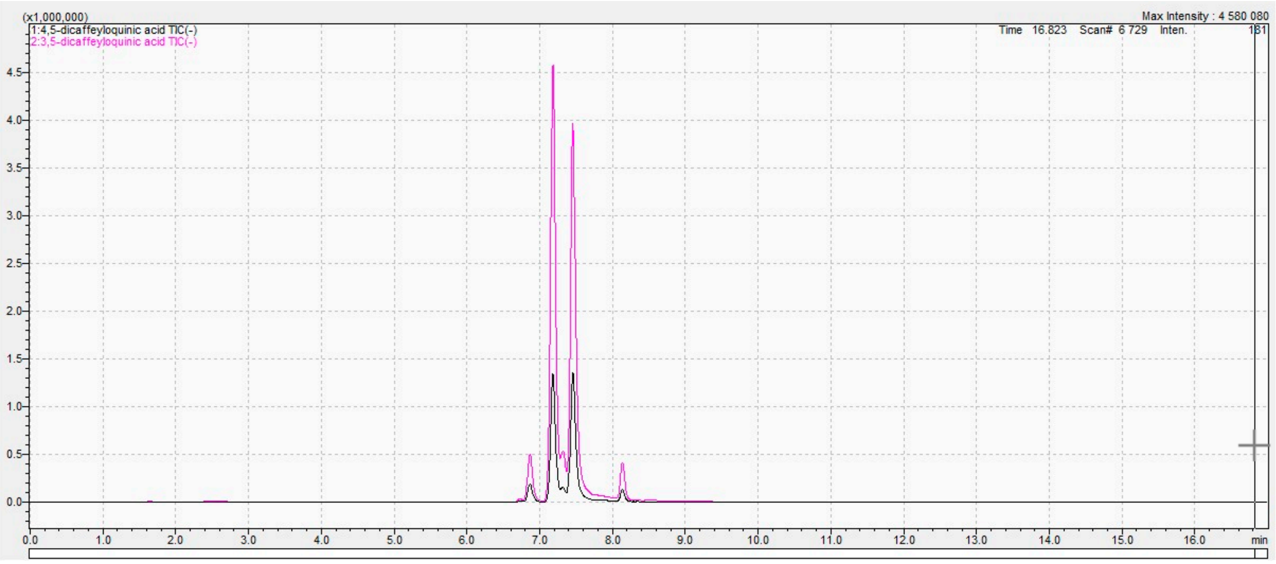

Figure S2. LC-MS chromatograms of 3,5-dicaffeoylquinic acid and 4,5-dicaffeoylquinic acid detected in *Ilex aquifolium* leaf extract.

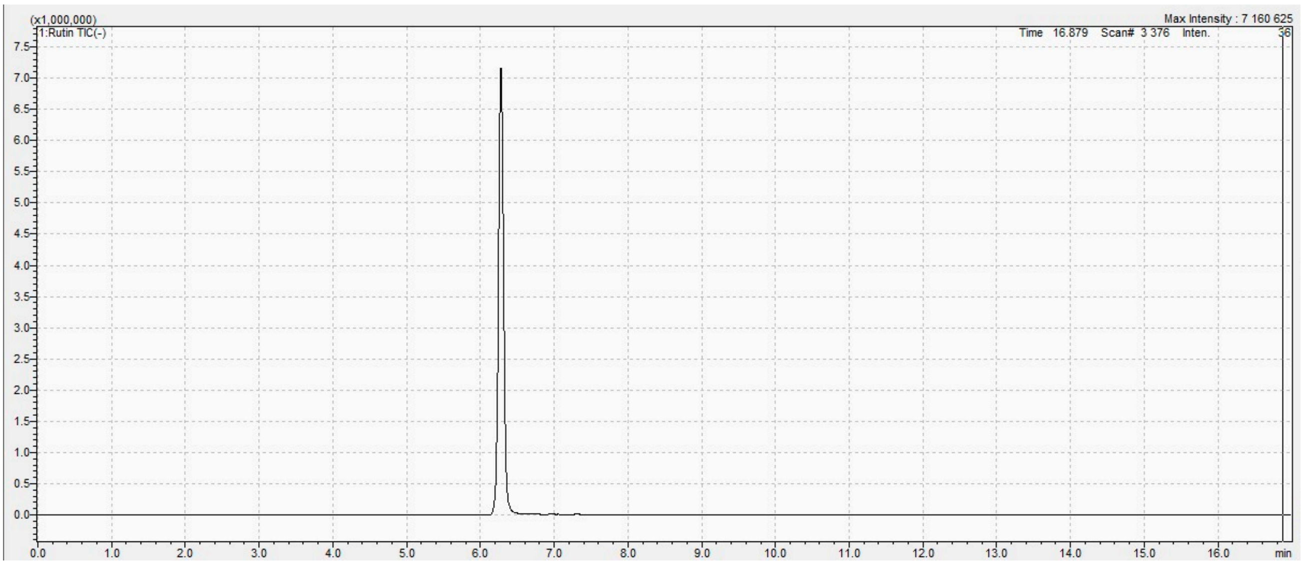

Figure S3. LC-MS chromatogram of rutin detected in *Ilex aquifolium* leaf extract.

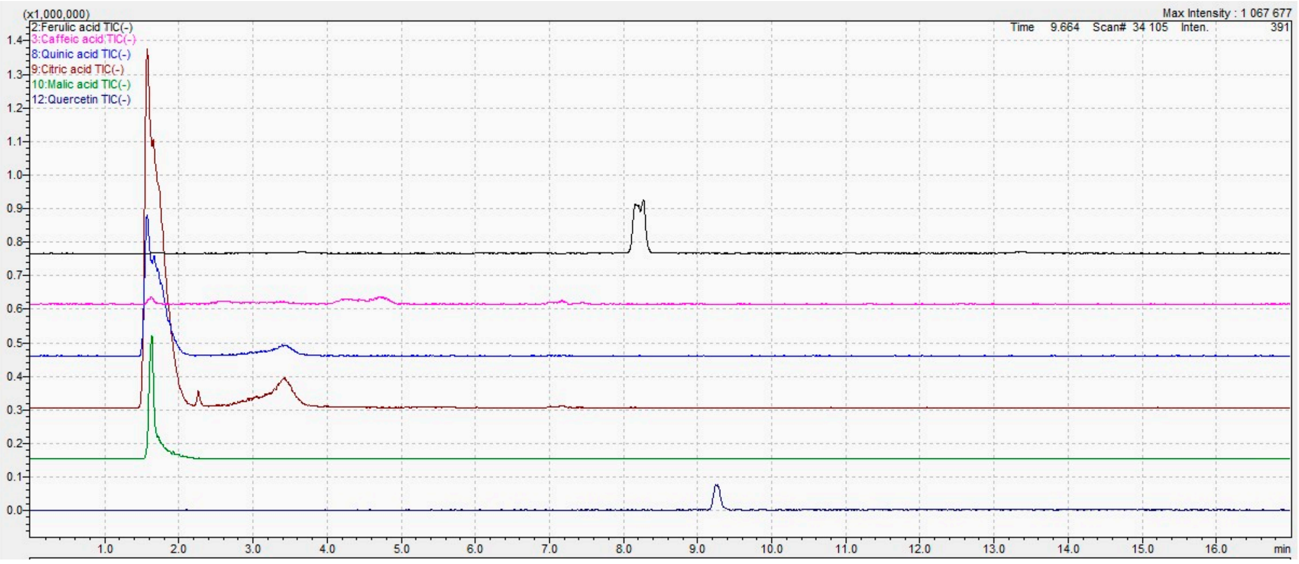

Figure S4. LC-MS chromatograms of ferulic acid, quinic acid, citric acid, malic acid, and quercetin detected in *Ilex aquifolium* leaf extract.
